# Autoimmune Inflammation and Insulin Resistance: Hallmarks So Far and Yet So Close to Explain Diabetes Endotypes

**DOI:** 10.1007/s11892-021-01430-3

**Published:** 2021-12-13

**Authors:** Alessandra Petrelli, Anna Giovenzana, Vittoria Insalaco, Brett E. Phillips, Massimo Pietropaolo, Nick Giannoukakis

**Affiliations:** 1 San Raffaele Diabetes Research Institute, IRCCS Ospedale San Raffaele, Milan, Italy; 2 Vita-Salute San Raffaele University, Milan, Italy; 3 Institute of Cellular Therapeutics, Allegheny Health Network, Pittsburgh, PA USA; 4 Division of Diabetes Endocrinology and Metabolism, Department of Medicine, Baylor College of Medicine, Houston, TX USA

**Keywords:** Insulin resistance, Autoreactive T cells, Autoimmune inflammation, Diabetes endotypes, Adipose tissue

## Abstract

**Purpose of Review:**

Diabetes mellitus can be categorized into two major variants, type 1 and type 2. A number of traits such as clinical phenotype, age at disease onset, genetic background, and underlying pathogenesis distinguish the two forms.

**Recent Findings:**

Recent evidence indicates that type 1 diabetes can be accompanied by insulin resistance and type 2 diabetes exhibits self-reactivity. These two previously unknown conditions can influence the progression and outcome of the disease. Unlike most conventional considerations, diabetes appears to consist of a spectrum of intermediate phenotypes that includes monogenic and polygenic loci linked to inflammatory processes including autoimmunity, beta cell impairment, and insulin resistance.

**Summary:**

Here we discuss why a shift of the classical bi-modal view of diabetes (autoimmune vs. non-autoimmune) is necessary in favor of a model of an immunological continuum of endotypes lying between the two extreme “insulin-resistant” and “autoimmune beta cell targeting,” shaped by environmental and genetic factors which contribute to determine specific immune-conditioned outcomes.

## Introduction

Diabetes mellitus is a metabolic disease clinically united by acute and chronic hyperglycemia. Two major categories, type 1 (T1D) and type 2 (T2D) diabetes, are the most prevalent. They are distinguished by (i) timing of disease onset, (ii) genetic predisposition, and (iii) clinical phenotype. T1D is an autoimmune process that begins years prior to the clinical onset resulting in the autoimmune-mediated, selective impairment of the pancreatic beta cells. T2D, instead, is characterized by peripheral insulin resistance (IR) and beta cell insufficiency [1]. To compensate for IR, beta cells are compelled to produce more insulin establishing a stress condition that eventually imperils their functional responses to hyperglycemia [1]. Even as these two conditions converge clinically at hyperglycemia, accumulating evidence indicates that they share many more features than originally thought. Considering that latent autoimmune diabetes of adults (LADA) presents features of T1D and T2D, that IR is seen in overweight T1D patients [2], and that some T2D patients exhibit pancreatic autoimmunity [3•], it may be time to re-think the separation of T1D and T2D and, instead, think of the conditions along immunoetiopathogenic lines.

Accumulating evidence also reveal an under-appreciated role for T cells driving IR in T1D, in addition to their known role in their selective targeting of beta cells. In this perspective, we discuss diabetes immunoetiopathogenesis from a novel viewpoint that T1D and T2D represent a continuum of immune-mediated responses to physiologic attempts to maintain a glucocentric metabolic homeostasis, where “endotypes” shape and define the continuum between autoimmunity and IR.

## Heterogeneity of Diabetes Mellitus: the Emerging Role of Precision Medicine

T1D and T2D are heterogeneous diseases caused by a complex interplay of genetic, epigenetic, and environmental factors. Although many genes predisposing to T1D and T2D are known, disease risk genotypes of individual patients span a large spectrum. T1D is an autoimmune disease with a small number of genes having large effects, (i.e., HLA) and a large number of genes having small effects. Risk of T1D progression is mainly attributed to specific HLA DR/DQ alleles [4]. The heterogeneity of T2D is even more complex given the large number of genetic variants found to be associated with risk or protection of this condition. To date, association of at least 120 variants with T2D risk and progression has been replicated [5], with genetic evidence supporting a unified risk model for different diabetes variants [6].

A number of environmental factors are thought to trigger and/or influence the severity of the autoimmune and the inflammatory attack on the pancreatic beta cells, and the specific immune mechanisms operative in individual patients appear to be variable [4]. At diagnosis, patients with T1D, LADA, or T2D can present with marked hyperglycemia, insulin resistance, and ketoacidosis, with asymptomatic or mild postprandial hyperglycemia. The rate of decline in beta cell function prior to and after diagnosis of these conditions is also extremely variable.

What is rapidly changing in the practice of medicine is the ability to characterize and understand human biological variation through assessment of the genetic and metabolic state of an individual, generating and understanding relevant data to develop likely disease categories, and implementation of preventive and treatment strategies tailored to specific conditions. Each form of diabetes has features that permit application of precision medicine as well as barriers to its implementation [7]. Diabetes caused by single gene defects (monogenic) can be characterized and targeted therapies are particularly effective. In T1D, islet autoantibodies and genomic risk have been identified as potential biomarkers of risk, facilitating immune intervention trials and pre-onset monitoring to reduce risk of severe complications and aiding in detection of environmental triggers. In T2D, multiple biomarkers and genetic variants have been shown to alter risk of disease, revealing new biological pathways and providing potential drug targets.

The concept of “endotype” is an etiopathological pathway that can inform targeted therapy [8]. This concept is gaining traction and hopefully in the near future we should be able to identify the endotype of the patient in the form of a tailored therapy for diabetes mellitus based on the etiopathogenesis and the natural history of this syndrome.

## Autoimmune Inflammation of Insulin-Sensitive Tissues in Obesity-Associated Diabetes

Obesity-associated diabetes has been mechanistically linked to a low-grade chronic inflammation induced by excessive and prolonged caloric intake. Metabolic dysregulation occurs in the adipose tissue leading to the release of pro-inflammatory molecules, such as free fatty acids (FFA), tumor necrosis factor ($\mathrm{TNF}\alpha$), interleukin 6 (IL-6), macrophage chemoattractant protein (MCP-1), inducing IR by inhibiting insulin signaling and recruiting other pro-inflammatory leukocytes, including M1 macrophages [1]. However, several other leukocyte populations, such as dendritic cells (DC), neutrophils, invariant-NK-T cells, and T and B cells, are altered in circulating frequency and phenotype in obesity and have been implicated in the development of IR.

T cells appear to be involved in the very early stages of the pathological cascade leading to IR. After 5–6 weeks of high-fat diet (HFD), CD4+ and CD8+ T cells infiltrate adipose tissue prior to macrophage accumulation and temporally parallel to the onset of hyperglycemia [9]. Upregulation of T helper 1 (Th1) surface proteins associated with a pro-inflammatory state in adipose tissue occurs by 2 weeks of the initiation of a HFD regimen [10]. These findings, supportive of the hypothesis that T cells are relevant in the initiation of IR, are further reinforced by the observation that amelioration of insulin sensitivity can be achieved using T cell targeting immunotherapy [9]. T cell alterations, namely increased frequency of pro-inflammatory phenotype, can be found in T2D patients [11•], although it is not clear if they are involved in the initiation or the maintenance of IR.

Since T cells are MHC-dependent for their target, it is of interest to determine if those T cells enriched at the target site of inflammation in T2D are antigen-independent bystanders, or if they are selected in a T cell receptor (TCR)-dependent manner. A growing body of evidence suggests that, in T2D, adipose tissue and/or pancreas may be the site of self-antigen-dependent priming of T and B lymphocytes which, in turn, actively participate to the metabolic decline.

### Visceral Adipose Tissue

Visceral adipose tissue (VAT) depots, including omental and mesenteric adipose tissue, contribute to the development of cardiovascular diseases (CVD) and T2D [12]. It is becoming evident that the TCR repertoire of T cells infiltrating the VAT of obese mice is less heterogeneous than in lean mice. Indeed, restriction of the complementarity-determining region 3 (CDR3) of the TCR in CD8+ T cells [13•] and of TCR-Vα and TCR-Vβ repertoire in CD4+ T cells infiltrating the VAT of obese mice has been described [14, 15]. This would be suggestive of T cell clonal expansion upon antigen recognition. Heterogeneity of the TCR repertoire has yet to be investigated in the VAT of patients with T2D; however, in Pima Indians, a population with high incidence of obesity and T2D, there is a shortened circulating CDR3 and higher frequency of TRBV7-8 [14]. Of note, it has been suggested that reduced length of CDR3 region confers higher risk of autoimmune disease [16].

The work of Deng et al. [10] provides mechanistic insights on how T cells are primed by adipocytes which, under stress conditions, can become capable of antigen presentation. Indeed, expression of major histocompatibility complex class II (MHC-II) on adipocytes is upregulated in obesity, and co-culture of adipocytes from obese donors with splenic CD4+ T cells induces T cell expansion and production of IL-2 and IFN-γ. Furthermore, T cell priming is antigen-dependent as the genetic deletion of the MHC-II complex in adipocytes reduced IR. Antigenicity of adipocytes was confirmed by another report showing that expression of MHC-II is higher in large versus small adipocytes [17]. Collectively, these data suggest that, in a condition of hyperinsulinism, such as obesity and T2D, adipocytes can acquire antigen presenting capacity with consequent induction of T cell expansion.

The presence of autoimmune inflammation in the VAT is also highlighted by the role of autoantibodies. Transfer of IgG from obese mice to B cell-depleted recipients leads to dysglycemia, suggesting that autoantibodies are pathogenic in the context of IR [18]. Furthermore, a recent report identified IgG antibodies specific for adipocyte-derived antigens (e.g., signal transduction molecules, metabolic and DNA repair enzymes, hormones, histones) in the plasma and in the fat of obese patients [3•].

These data indicate that alteration of antigen recognition and induction of autoantibodies in the VAT is associated with T2D, which strengthens the hypothesis that adipose-specific autoimmunity may be mechanistically relevant in the development of IR.

### Pancreatic Beta Cells

T2D-associated beta cell damage is thought to be the end result of metabolic stress in combination with localized inflammation mediated primarily by infiltrating macrophages. However, circulating islet-reactive T cells have been found in patients with T2D, and this was associated with a more compromised beta cell function compared to patients without islet-reactive T cells [19]. Notably, T cell autoreactivity and production of autoantibodies develop after the onset of T2D and are associated with a more rapid decline of beta cell function [20]. Another study confirmed the presence of circulating islet-reactive CD4+ T cells in T2D patients, while CD8+ T-cell reactivity to islet antigen was found to be a unique feature of T1D [21]. It is possible that the higher demand of beta cells due to IR promotes beta cell stress and eventual death, antigen presentation, and generation of islet-reactive T cells. Islet reactivity may be not as destructive as in T1D, probably due to the lack of autoreactive CD8+ T cytotoxic cells. As a matter of fact, T (and also B) cells are part of the peri-insulitis and exocrine pancreas infiltrate in T2D patients [22]. It is possible, however, that antigen-specific lymphocytes do not stop in the pancreas but rather recirculate to insulin-sensitive tissues leading to an “infectious inflammatory state” that ultimately induces IR.

An alternative hypothesis is that T cells activated in the VAT of obese patients may contribute to beta cell death either by migrating to the pancreas or by releasing exosomes harboring soluble mediators targeting beta cells. This is supported by the evidence of a cross-talk between immune cells, adipose tissue, and beta cells with both T cells and adipocytes releasing exosomes containing specific microRNAs regulating survival and function of pancreatic beta cells [23, 24].

When considered together, two equally likely hypothesis can reconcile the presence of autoimmunity in T2D and IR in T1D: (i) adipose-reactive T cells generated in the VAT migrate to other metabolically active tissues where they induce IR or (ii) islet-reactive T cells generated in the pancreas, migrate to VAT and other metabolically active tissues where they induce IR either by sustaining chronic tissue inflammation or by targeting protective elements for the development of IR.

## Insulin Resistance as a Feature of Autoimmune Diabetes

IR can be defined as the impaired sensitivity of peripheral tissues to the biological actions of insulin, whether they are mediated by the insulin receptor or insulin/type 1 insulin growth factor hybrid receptors. IR also causes stress to the insulin producing beta cells which, as a consequence of the IR state, are required to produce more insulin to compensate for hyperglycemia. This establishes a vicious circle where IR stresses beta cells and where increased insulin finds more resistance to its actions in the otherwise insulin-sensitive tissues and organs. In T1D, especially in overweight patients, this condition is even more pronounced as a consequence of pharmacologic insulin replacement [25].

Using the sensitive hyperinsulinemic-euglycemic clamp approach, a number of studies showed the presence of IR in T1D patients [26]. While exogenous insulin administration may be one of the drivers of IR [27], there are other underlying mechanisms that are already in place in establishing IR independently of exogenous insulin. Studies in pre-symptomatic T1D patients show that relatives of patients with T1D who most rapidly developed the disease had greater insulin resistance for their level of insulin secretion [28]. Furthermore, a recent report demonstrated that normal weight at-risk individuals with elevated autoimmunity (2 or more anti-islet antibodies) have higher levels of insulin resistance compared to healthy controls [29••]. However, no significant relationships between IR and progression from one to multiple autoantibodies or to T1D were found in the TrialNet cohort [30]. Another study showed that only in a fraction of relatives with T1D, meaning autoantibody-positive relatives in whom insulin secretion is markedly reduced, insulin resistance accelerated progression to T1D [31]. This is in line with reports showing that excess body weight is associated with increased risk to develop T1D [32]. On the other hand, mice of the non-obese diabetic (NOD) strain, which develop autoimmune T1D, fed a HFD that promoted IR, are paradoxically protected from developing diabetes [33]. The investigators proposed that immunoregulation is induced by gut microbiome-mediated generation of regulatory T cells (Tregs), suggesting a crucial role of diet in the development of autoimmune diabetes.

### Relevance of Liver Insulin Resistance in Autoimmune Diabetes

While peripheral IR has been characterized in many human studies, less understood is hepatic IR in T1D. Regulation of peripheral glucose concentrations and restraint of hepatic glucose production in T1D requires large concentrations of exogenous insulin [34]. However, the intraportal concentrations of insulin required to restrain hepatic glucose production are much lower in T1D than what is needed for peripheral glucose uptake and utilization. This creates a therapeutic conundrum, where, on the one hand, too much insulin, even though it responds to the need for peripheral glucose uptake and utilization, causes and exacerbates IR, while on the other hand, a low intraportal insulin concentration would be better for optimal suppression of glucose production.

In what ways then can liver IR and inflammation be used as platforms to clarify and establish endotypes of diabetes? Non-alcoholic fatty liver disease (NAFLD), which is characterized by the excessive storage of lipid and IR in liver, is oftentimes a comorbid condition in T2D that occurs in as many as 22% of adults with T1D [35] implicating the presence of liver IR in these patients. The T2D drug metformin works in part by increasing liver insulin sensitivity and reducing hepatic glucose production. Metformin use as an adjunctive to manage NAFLD has been tested in clinical trials. Data from recent T1D clinical trials, however, could not establish a clear benefit, but did show reduced insulin requirements, decreased circulating LDL, and indicated protection from atherosclerosis [36]. At the same time, NAFLD and T1D development is associated with a type 1 interferon response that drives T helper 1 cell expansion [37]. What has been largely unexplored is if the type 1 interferon response in the liver is capable of inducing auto-immune responses in T2D, where as many as 10% of patients present with autoantibodies. Beta cell endoplasmic reticulum (ER) stress, inflammation, and apoptosis are indicative of T2D [38] and when paired with a type 1 interferon response could establish conditions for the production of stress-induced “neoantigens” that act as self-antigens. NAFLD could therefore provide a diagnosable clinical context of shared IR and/or auto-immunity mechanisms in both T1D and T2D.

## Where and How Self-reactive T Cells Can Be Primed in Insulin Resistance

**Figure 1:**
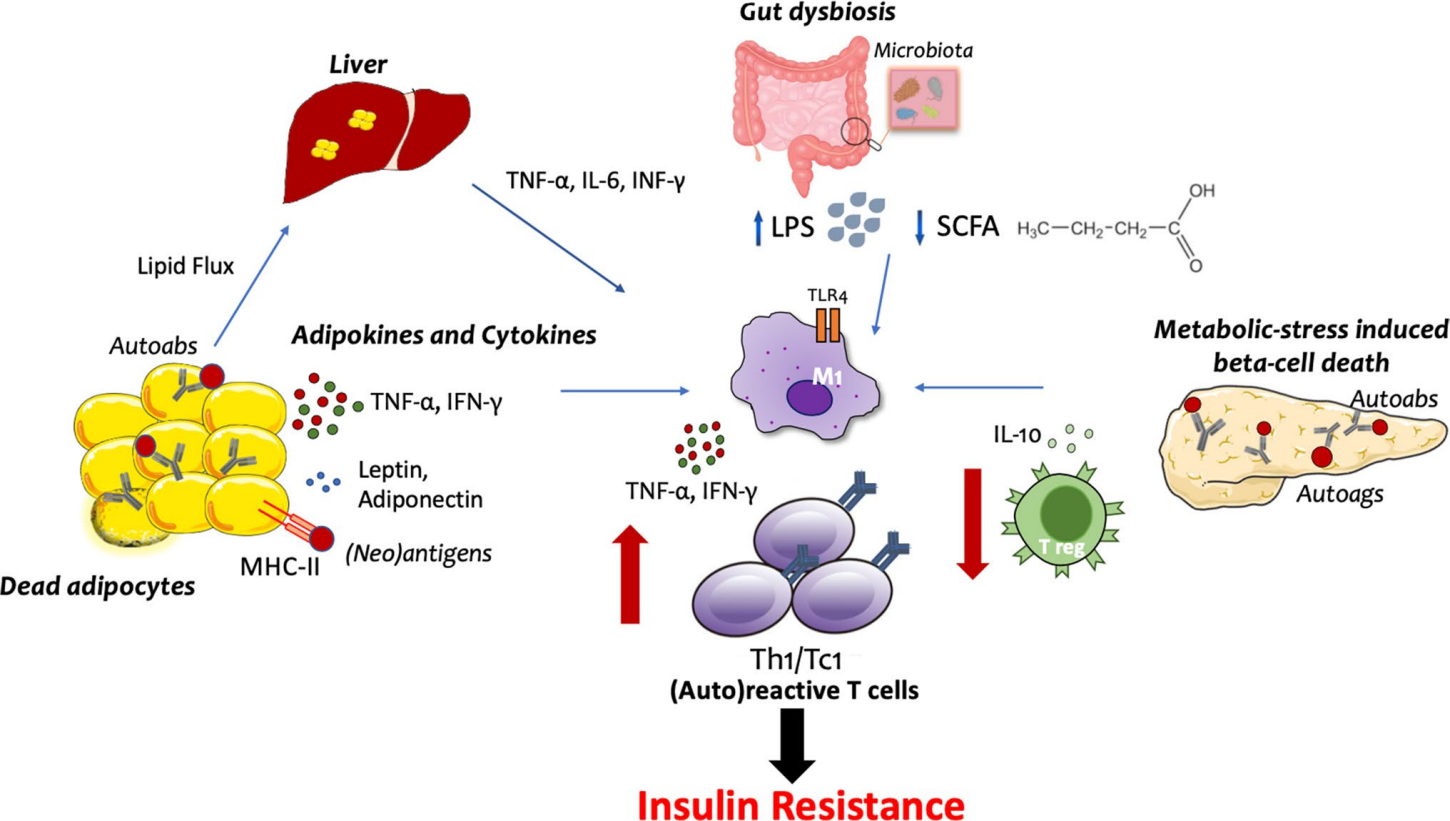
Potential mechanisms of engagement of autoreactive T cells in insulin resistance. Schematic view of the proposed mechanisms involved in the generation of autoreactive T cells in the context of insulin resistance: (i) In the visceral fat, accumulation of fat depot induces adipocyte death and release of pro-inflammatory soluble hormones and cytokines. This is accompanied by the upregulation of MHC-II on adipocytes which turn into antigen-presenting cells; (ii) Intestinal dysbiosis, by promoting translocation of lipopolysaccharide (LPS) and bacteria metabolites in the systemic circulation, induces autoreactive immune responses in the gut as well as in other tissues; (iii) In the pancreas, metabolic-stress and inflammation induce beta cell death with the generation of conventional and neo-antigens; (iv) Local and systemic inflammation is fostered by the excessive accumulation of lipids in the liver. All these pathways lead to TLR-4-mediated activation of tissue resident macrophages, enhanced presentation of autoantigens, and consequent development of (auto)reactive T cells with a type 1 T helper (Th1) or cytotoxic T cell (Tc1) phenotype. This is accompanied by the reduction of regulatory T cell subsets and cytokines, such as Treg and IL-10, which contribute to the development of resistance to the insulin action in insulin-sensitive tissues. Autoags: autoantigens; Autoabs: autoantibodies

Multiple mechanisms may be involved in the generation of autoreactive T cells in the context of IR. In addition to lipid metabolism, adipocytes perform a number of local and systemic activities, including the release of a variety of cytokines and adipokines with endocrine and immunoregulatory functions that alter number, differentiation, and function of T cells [39]. The net effect is a skewing toward a type 1 T helper (Th1) phenotype. The metabolic imbalance induced by obesity may also favor the emergence of autoreactive T cells via the impairment of mechanisms of immunological tolerance. Indeed, nutritional overload perturbs the mTOR pathway leading to impaired differentiation of Tregs [40]. Furthermore, our group showed that VAT-derived CD4+ conventional T cells and CD8+ T cytotoxic cells from obese patients with dysglycemia are resistant to suppression, thus confirming that counterregulatory mechanisms are compromised in the context of IR [11•]. Presentation of (neo)antigens due to adipocyte and/or beta cell stress and death, accompanied by intestinal dysbiosis and liver-derived inflammation, concurs to the engagement of adaptive immunity in the context of IR (Figure 1).

### Adipocyte Apoptosis

Hyperplasia and hypertrophy of adipocytes, accompanied by adipose tissue hypoxia and adipocyte death, are features of obesity. Dying adipocytes induce recruitment of macrophages that surround dead adipocyte creating the so-called crown-like structure [41]. The finding of autoantibodies directed toward adipocyte antigens in T2D patients corroborates the hypothesis that adipocyte death can be a source of antigens driving T cell priming in IR. Indeed, inhibition of adipocyte death by knocking out the pro-apoptotic protein Bid prevented IR in a preclinical model of obesity [42]. However, adipocyte death was shown to occur after 8 weeks of a HFD, once glucose metabolism is already impaired [43]. Furthermore, the pathological effect of autoantibodies is stronger when mice have an advanced stage of diabetes [18], suggesting that autoimmunity is a dynamic process likely fueled by continuous adipocyte death. Although adipocyte apoptosis is observed in human IR [42], autoantigens may not necessarily derive from dying adipocytes; indeed, in vitro presentation of adipocyte-derived antigens occurs with live cells [10]. Excessive expansion and inflammation of adipose tissue resulting in adipocyte cell death may favor the generation of autoreactive lymphocytes. The dynamics of these events leading to the development of IR have yet to be established in human pathology.

### (Neo)antigen Presentation

As described earlier (Section “Visceral Adipose Tissue”), in obese mice, antigen-specific T and B cells are activated in response to adipocyte-derived antigens [3•, 10]. Post-translational modification (PTM) of native antigens, alternative splicing, and defective ribosomal products may generate novel epitopes which are identified as non-self [44]. It is conceivable that in T1D and LADA, novel neuroendocrine neoantigens may enhance T cell immunogenicity as the attendant hyperglycemic and pro-oxidative metabolic milieu includes abnormal glycosylations, deamidation, and oxidative damage to proteins within pancreatic beta cells. Metabolic stress-induced adipocyte death inside a pro-inflammatory milieu may foster the generation of conventional antigens or neoantigens by adipocytes, similarly to what is observed in T1D [45]. ER stress on both beta cells and adipocytes occurs in response to the metabolic stress induced by obesity [46]. By promoting abnormal PTMs, obesity may foster the generation of neoantigens and, in turn, the development of autoreactive responses to these epitopes. VAT-derived MHC class I-associated immunopeptidome isolated from lean and obese mice shows that some MHC I-restricted peptides, such as LDHA237-244, are present exclusively in obese VAT and exhibited dose-dependent immunogenicity to induce CD8 T cell responses [47••]. These data support the hypothesis that T cells can be primed in the VAT by adipocyte-derived antigens generated as a result of PTMs, thus representing a crucial event in obesity-induced VAT inflammation.

### Gut Dysbiosis

Gut dysbiosis has been linked to T2D and obesity. Increased abundance of *Bacteroidetes* and decreased abundance of *Firmicutes* strains observed in T2D and obesity result in lower levels of microbiota-derived metabolites, such as short-chain fatty acids (SCFA), known to promote Treg differentiation and production of the anti-inflammatory cytokine IL-10 [48]. Gut dysbiosis has also been associated with islet autoreactivity, with the loss of gut barrier integrity leading to translocation of bacterial products that stimulate islet-reactive T cells through a TCR-mediated mechanism [49]. Dysbiosis occurring in obesity and T2D may similarly trigger autoimmunity. Indeed, HFD, by increasing gut barrier permeability and intestinal absorption of antigenic material [50], may induce autoimmune responses especially in tissues in close proximity of the gut, such as VAT. Triglycerides promoted intestinal absorption of the protein antigen ovalbumin (“OVA”) and its localization in mesenteric adipose tissue, which was associated to in situ accumulation of CD4+ T cells [51]. Increased intestinal permeability also allows bacterial products, such as lipopolysaccharide (LPS), to translocate in the systemic circulation, reach the VAT, and activate local immune responses via high-affinity binding to TLR4, eventually leading to IR [52]. The role of the gut microbiome in IR has been partially addressed in humans. Wu et al. [53] showed that metformin-treated T2D patients exhibited improved IR, change in gut microbiome, and increased levels of SCFA. However, further studies are required to establish whether the targeting of IR by metformin is mediated by the restoration of a healthy microbiome.

Accumulation of bacterial LPS may contribute to metabolic stress/cell death in adipose tissue or pancreas with enhanced exposition of autoantigens and consequent development of T cell autoreactivity. Autoreactive T cells then can migrate to the circulation with consequent extravasation in metabolically active organs. Therefore, regulation of immune homeostasis by gut dysbiosis, by fostering the development of the ideal milieu for the generation of autoreactive immune responses, may possibly result in the initiation of IR.

## Implications for Therapy

Given the growing prevalence of IR in T1D, especially in overweight individuals, increasing insulin dose may not be the best approach as it can very likely exacerbate IR.

Insulin sensitization using metformin was the obvious adjunctive approach; however, unlike in T2D, any possible benefit of this drug has been small in T1D [54]. Oral insulin and insulin analogs that exhibit preferential hepatic bioavailability are another possible therapy that can confer a better insulin balance between portal and peripheral circulation. However, there are issues of limited bioavailability [55] and concerns about potential pathologic hepatic effects. Other approaches to improve insulin sensitivity in T1D include glucagon-like peptide-1 receptor agonists and liver-selective glucokinase activators. Glucagon receptor antagonists can lower insulin needs while improving glucose time-in-range and decreasing hypoglycemia times [56]. These agents however, other than being highly experimental, may interfere with exogenous glucagon in cases of severe hypoglycemia and can promote weight gain. Liver-selective glucokinase activators are very promising based on available data, as they reduce hypoglycemia while lowering peripheral glucose concentrations with lower insulin requirements [57].

To halt the low-grade systemic inflammatory state that is not of autoimmune nature, anti-inflammatory approaches such as CXCR2 antagonists [58] could be helpful adjunctive treatments in T1D. Of note, targeting TNF-α in new onset T1D [59] improved endogenous insulin production and lower insulin requirement, suggesting a potential effect on peripheral insulin sensitivity. No data are available on the effect of T cell modulation on IR in the prevention and treatment of T1D in humans. However, immunotherapy has the potential to be effective in the context of IR as administration of abatacept, targeting T cell co-stimulation, has improved insulin sensitivity in patients with rheumatoid arthritis [60]. To elucidate whether T cell modulation interferes with IR, insulin sensitivity indices should be assessed in clinical trials with T cell targeting compounds in T1D. In line with this, clinical trials should be designed to elucidate whether immunotherapy has an effect on the modulation of IR and T2D.

## Diabetes as an Immunologic Continuum

Diabetes has long been considered a multifaceted disease due to the heterogeneity of clinical manifestations, different aetiologies, and genetic factors contributing to its development [61]. Clustering diabetes into distinct subgroups, or “endotypes”, based on specific pathophysiological processes would impact the clinical management of the disease. In the effort of accounting all the different factors that contribute to T2D development, McCarthy has proposed the “Palette” model to predict the trajectory of metabolic derangement starting from specific pathological defects observed in these patients [62]. We, likewise [8], have introduced the concept of endotypes in the field of T1D to highlight the presence of multiple mechanisms underlying T1D development.

**Figure 2:**
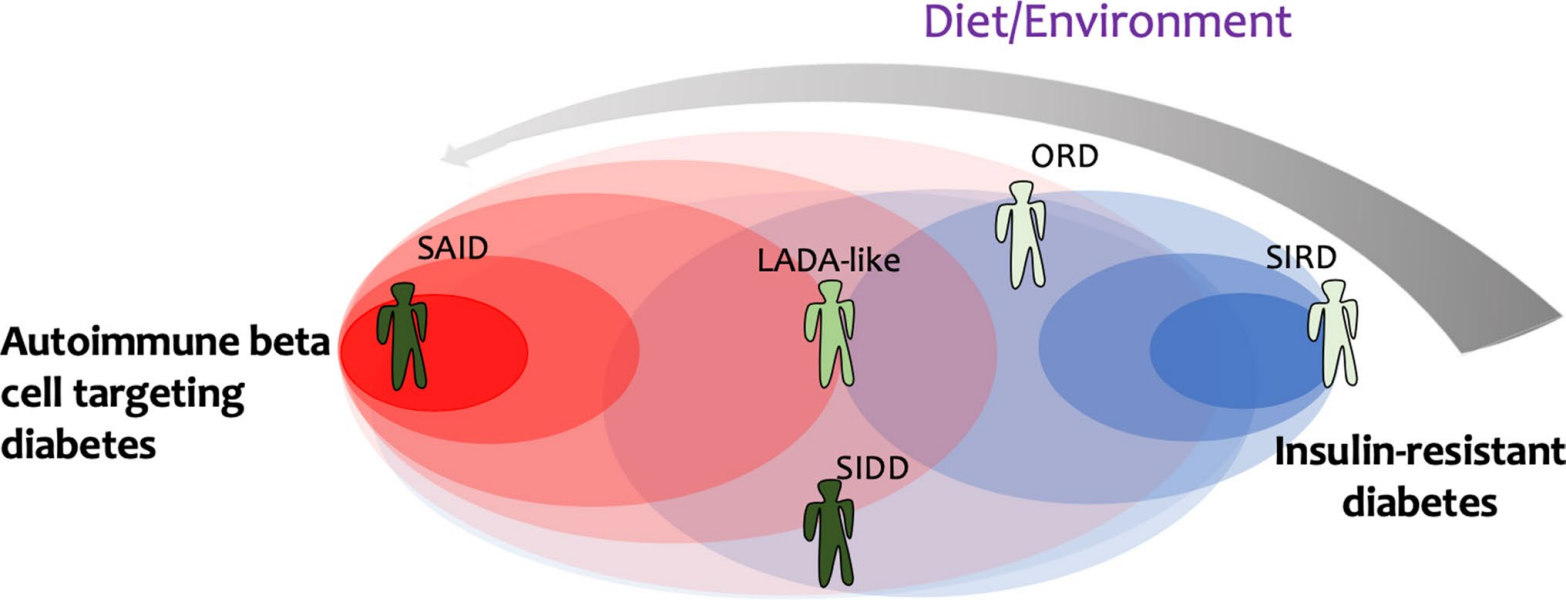
Application of the “continuum model” to define diabetes endotypes. Spectrum of diabetes endotypes ranging from “autoimmune beta cell targeting” (red) to “insulin-resistant” diabetes forms (blue). Color intensity indicates the magnitude of islet autoimmunity and insulin-resistance components, red and blue respectively. The impact of diet/environment on diabetes endotypes is shown by the gray arrow (the larger and darker the arrow, the stronger the effect). Each individual represents a specific endotype, whose position is the result of a cumulative overlay of multiple factors (islet autoimmunity, insulin-resistance, diet/environment). The shades of green of each individual indicate the weight of genetics in determining the endotype, dark green refers to a stronger effect than light green. We used the clusters identified by Ahlqvist et al. (63) as endotypes that may fit our model, i.e., the Severe Autoimmune Diabetes (SAID) endotype, Severe Insulin-Resistance Diabetes (SIRD), Severe Insulin-Deficient Diabetes (SIDD), and Obesity-related diabetes (ORD). This cluster analysis approach may be enriched by other disease variants, such as the “LADA-like” endotype, that lies in the middle and shows intermediate traits

In this perspective, we have highlighted that diabetes often results in the admixture of pathological mechanisms of T1D and T2D, suggesting that the distance between their aetiologies is smaller than originally thought, and that they may represent the extreme manifestations of a continuum immunological process. In light of this consideration, we propose a novel model to interpret diabetes, in which the disease is not formally distinguished into T1D or T2D forms but is rather thought of as an immunologic continuum shaped by different endotypes between the two disease extremities (beta cell targeting autoimmunity and immune-sensitizing metabolic disruption resulting in IR). This continuum model is illustrated in Figure 2 and is based on a common motif whose platform is the immune system. As a simplification, the model here described is based on disease clusters identified by Ahlqvist et al. [63]: the Severe Autoimmune Diabetes (SAID) endotype, featured by islet autoimmunity and ketoacidosis, lies at the “autoimmune beta cell targeting” extremity, while the Severe Insulin-Resistance Diabetes (SIRD) would correspond to the “insulin-resistant” endotype, in which high levels of IR and low autoimmunity are present. Severe Insulin-Deficient Diabetes (SIDD) is far from the diet/environment arrow and has a high genetic component. Obesity-related diabetes (ORD), featured by elevated BMI and intermediate levels of IR, is tightly associated with diet and environmental triggers. Here, we propose that a certain endotype is defined by the combination of specific factors such as autoimmunity, insulin resistance, genetic, and environmental factors. However, this is still a simplification. Indeed, in the “insulin-resistant diabetes” extreme of the model, different endotypes are likely to co-exist, which may differ for the way the immune system induces insulin resistance, such as the dynamics and tissue localization of (autoreactive?) T cells. Moreover, we reasoned that genetic and environmental factors may dictate the specificity of autoantigens, the profile of autoreactive T cells, and the type and magnitude of autoimmune responses, thus skewing the endotypes toward a strong autoimmune versus insulin-resistant component.

## Conclusions

In the view of emerging personalized therapies, endotype-specific features should be part of a treatment approach. As IR and autoimmunity are often part of the clinical course of diabetes, clinical trials are needed to explore the benefit of diet/healthy lifestyle, insulin-sensitizing agents, and immunomodulating therapies on distinct diabetes endotypes. Here we propose a model in which endotypes are defined by the combination of autoimmunity and insulin resistance representing the two extremities of an immunological continuum model in which genetic and environmental factors contribute to determine the specificity of the immune response.

With this model, we do not aim to underestimate the importance of the diet in the induction of metabolic dysfunction or the role of beta cell fragility, nor we want to exclude monogenic diabetes or downsize the role played by the innate immune system in the development of different endotypes of diabetes. The effort of dissecting specific mechanisms that contribute to the definition of new endotypes should be pursued to develop precision medicine as well as to prevent or mitigate the onset of diabetes in at-risk subjects.

## Declarations

### Guarantor Statement

As guarantor of this work, AP assumes responsibility for the originality and integrity of the content reported in the manuscript and the decision to publish.
